# Comparison Between the Original PTGI and the PTGI-SF in a Large Sample of New Mothers

**DOI:** 10.3390/bs15121641

**Published:** 2025-11-28

**Authors:** Orit Taubman – Ben-Ari

**Affiliations:** School of Social Work, Bar-Ilan University, Ramat Gan 5290002, Israel; taubman@biu.ac.il

**Keywords:** personal growth, posttraumatic growth, perinatal, mothers

## Abstract

The first objective of this study is to review and synthesize research from the past fifteen years on variables associated with personal growth (or post-traumatic growth, PTG) during the transition to motherhood, and to revisit key questions using a uniquely large dataset; the second objective of the study is to compare findings across different PTG scoring methods, to determine whether the long and short forms of the Post-Traumatic Growth Inventory (PTGI) produce equivalent data and whether the five growth domain scores provide additional insights beyond the total scores. Data were aggregated from multiple previous studies, resulting in a large sample (*n* = 4641) of first-time mothers with infants up to 24 months old (M = 6.82 months, SD = 4.42). Analysis of the associations between demographic and childbirth characteristics and PTG scores revealed that in most cases, the original PTGI and the short version (PTGI-SF) exhibited similar associations with the background variables. The five PTGI domain scores of personal strengths, spirituality, new opportunities, and appreciation of life showed associations consistent with those observed for the two total scores. The findings reinforce and validate previous research, while also offering new insights. Notably, the results suggest that the PTGI-SF is highly useful, as it yields results comparable to those of the original, longer inventory.

## 1. Introduction

The transition to motherhood is widely recognized in the literature as a period of significant stress and anxiety, marked by profound life changes, a shifting sense of identity, and the adoption of new roles (Cowan & Cowan, 2000; Howard & Brooks-Gunn, 2009; Shapiro et al., 2020). Although this is viewed as a positive, usually expected, normative life event for women, and an important developmental milestone in adulthood (Katz-Wise et al., 2010; Lang et al., 2006), for many women the transition nevertheless entails various levels of existential anxiety and distress, as well as uncertainty and doubt (Koester & Petts, 2017; Yim et al., 2015; Bussolari & Goodell, 2009). The notion that women not only feel stress but also experience personal growth (also known as posttraumatic growth, PTG; Tedeschi et al., 2018) following the transition to motherhood was first introduced by Taubman – Ben-Ari and her team in 2010 and has since been the focus of substantial research efforts (Taubman – Ben-Ari, 2019). Tedeschi et al. (2018) define personal/posttraumatic growth as positive psychological transformation that is experienced as a consequence of struggle with significant life challenges.

The most widely used and reliable tool for assessing personal growth is the 21-item Posttraumatic Growth Inventory—PTGI, designed by Tedeschi and Calhoun (1996). The PTGI evaluates personal growth across five domains: Appreciation of Life, reflecting a heightened recognition of life’s value; Personal Strength, capturing an enhanced sense of resilience and self-reliance; New Possibilities, indicating the discovery of new opportunities; Relating to Others, reflecting enhanced interpersonal connections, greater openness with others, deeper emotional closeness, and increased compassion; and Spiritual Change, representing a deepened sense of faith or spirituality. The PTGI can be scored either as a total score of the overall holistic experience of personal growth, or across its five distinct domains. In 2010, a shorter form, the PTGI-SF was introduced, featuring ten items that generate a single total score (Cann et al., 2010).

The relevance and validity of the PTGI for measuring personal growth in mothers was established by Taubman – Ben-Ari et al. (2011) through multiple studies. First, new mothers were asked to respond to an open-ended question about changes in their lives following the birth of their first child. Their responses were analyzed and compared to the PTGI domains, revealing alignment with four of the five domains, excluding spiritual change, which did not emerge in their descriptions. This finding suggested that while the PTGI effectively captures positive transformations in motherhood, spirituality may not be a universally relevant domain.

To assess the PTGI’s applicability across different maternal experiences, a second study compared various subgroups, including first-time and experienced mothers, mothers of preterm and full-term babies, and mothers of twins versus singletons (Taubman – Ben-Ari et al., 2011). Across all groups, responses consistently aligned with the same four PTGI domains, reinforcing the tool’s universality and applicability for measuring maternal growth.

A third study introduced external validation by comparing mothers’ PTGI self-reports with independent assessments from their own mothers (Taubman – Ben-Ari et al., 2011). This approach, previously used in trauma research (Shakespeare-Finch & Enders, 2008; Weiss, 2002), revealed significant correlations, further supporting the PTGI’s convergent validity.

Since these foundational studies, ongoing research has explored factors contributing to personal growth in new mothers, with some factors shown as more consistently related to growth, while others showed more nuanced or mixed results (Taubman – Ben-Ari, 2019). The current study extends previous research, by integrating and analyzing data previously collected in Israel from both published and unpublished studies. This study aims to provide a comprehensive evaluation of the associations between the 21-item PTGI, its five domains and the 10-item PTGI-SF, on the one hand, and the sociodemographic and perinatal-related variables that have been explored in smaller and more limited studies to date, on the other.

Our main goals are to concisely review the knowledge that has been accumulated over the last fourteen years regarding variables related to personal growth in the transition to motherhood; to readdress several basic questions concerning associations between such variables and personal growth through the use of a uniquely large dataset; and to compare the findings from the different PTGI scores, using a large combined dataset, to assess whether the long and short forms of the PTGI provide similar results, as well as to determine whether using the separate scores from the five growth domains adds information to the two total scoring options.

### 1.1. Associations Between Demographic Variables and Personal Growth

Several demographic variables have been explored for their potential association with personal growth among new mothers. Younger **age** has been linked to higher levels of personal growth, both in first-time (primiparous) and non-first-time (multiparous) mothers (e.g., Ben-Yaakov & Taubman – Ben-Ari, 2021; Berman et al., 2021; Kaçan-Bibican et al., 2025; Sawyer et al., 2015; Taubman – Ben-Ari et al., 2012). This trend may be explained by the fact that younger age is associated with higher openness to learning and change (Tedeschi & Calhoun, 2004), or by the possibility that being a younger mother presents greater challenges and demands with fewer available resources, which may lead to greater stress, and thus to greater growth (Taubman – Ben-Ari et al., 2012).

Personal growth tends to be associated with lower **economic** status (Rozen et al., 2018), lower levels of **education** (e.g., Berman et al., 2021; Noy et al., 2015; Rozen et al., 2018) among both first-time and non-first-time mothers, and with **not being in a relationship** (Chasson & Taubman – Ben-Ari, 2021). Because being in a relationship is considered a source of comfort and support, single mothers may experience greater stress (Hertz, 2006), leading to higher personal growth. In addition, growth has been found to be related to **religiosity** in various ways, with longitudinal studies outside the context of motherhood indicating that faith is linked to greater PTG (Tedeschi et al., 2018). PTG has also been associated with better subjective physical **health** in general (Tedeschi et al., 2018), although this relationship has not been consistently observed among mothers (Noy et al., 2015; Taubman – Ben-Ari et al., 2019). Inconsistencies in smaller samples highlight the value of exploring these demographic variables within a large sample of mothers.

### 1.2. Associations Between Childbirth Characteristics and Personal Growth Following the Transition to Parenthood

Studies have also explored specific aspects of the childbirth experience and their relationship to personal growth, with mixed results. For instance, a study of Israeli mothers (Taubman – Ben-Ari et al., 2022) found that several factors related to childbirth—including labor duration, pain experienced during labor, and type of delivery—were not associated with personal growth. Another study conducted in the UK (Sawyer et al., 2012) found that the type of delivery predicted growth among women eight weeks after the birth of their first or second child. Specifically, mothers who underwent either elective or emergency cesarean sections, which are considered more threatening and even traumatic, reported higher levels of growth compared to those who had vaginal deliveries.

### 1.3. Association Between Stress-Related Perinatal Circumstances and Personal Growth

The process of personal growth is often driven by a certain level of stress (see for example, Stanton et al., 2006). However, distress arising from stress and growth can happen concurrently (Tedeschi & Calhoun, 2004; Tedeschi et al., 2018). Several studies have found that perinatal circumstances associated with higher stress correlate with higher personal growth. A series of Israeli studies found that giving birth to a premature single infant or premature twin infants was linked to higher levels of personal growth reported by mothers about one year postpartum (Noy et al., 2015; Taubman – Ben-Ari et al., 2010). In two longitudinal studies, mothers of premature babies reported greater personal growth than mothers of full-term babies at one month, six months, two years and even four years postpartum (Porat-Zyman et al., 2017; Spielman & Taubman – Ben-Ari, 2009; Taubman – Ben-Ari & Spielman, 2014; Taubman – Ben-Ari et al., 2019). Another study found that for mothers of premature infants, mental health improved over time, and this in turn was positively related to higher personal growth four years later (Porat-Zyman et al., 2019).

High-risk pregnancies as well as a history of miscarriage or stillbirth may also be experienced as particularly stressful (Isaacs & Andipatin, 2020; Krosch & Shakespeare-Finch, 2017). In a study among mothers who had high risk pregnancies, participants reported moderate to high degrees of PTG (Ha & Sim, 2015). In other studies, women who experienced a miscarriage or stillbirth reported moderate levels of PTG (Krosch & Shakespeare-Finch, 2017; Ryninks et al., 2022), with those who experienced a stillbirth reporting higher PTG than participants who experienced a miscarriage (Ryninks et al., 2022). Similarly, among women who underwent pregnancy termination for fetal abnormality, moderate PTG was observed in the domains of relating to others, personal strengths, and appreciation of life (Lafarge et al., 2017).

Yet another significant stressful perinatal factor is undergoing fertility treatment. However, studies have found no significant differences in personal growth between mothers who conceived following fertility treatments and those who conceived spontaneously (Noy et al., 2015; Porat-Zyman et al., 2017; Taubman – Ben-Ari et al., 2019; Taubman – Ben-Ari & Spielman, 2014). While fertility treatments may induce higher levels of stress during treatment and pregnancy (Braverman et al., 2024; Gameiro, 2024), they do not appear to have lasting effects on personal growth once parenthood is achieved (Abu-Sharkia & Taubman – Ben-Ari, 2024; Hjelmstedt et al., 2004). Finally, potentially stressful infant health factors, such as lower birthweight and lower Apgar scores were found to be associated with higher personal growth (Spielman & Taubman – Ben-Ari, 2009).

### 1.4. Association Between Previous Life Events or Special Circumstances and Personal Growth in the Transition to Parenthood

A relatively new path of investigation relates to previous stressful or traumatic events which may precipitate higher personal growth. For example, one study revealed that among Croatian but not among British first-time and non-first-time mothers, there was a correlation between higher levels of posttraumatic symptoms during pregnancy and higher reported growth following childbirth (Sawyer et al., 2015). This was explained through the idea that the Croatian women’s experience of war during their childhood affected their well-being, leading to posttraumatic reactions (Frančišković et al., 2007), which in turn triggered greater growth during the transition to motherhood. In another study, the loss of a parent up to seven years prior to becoming a mother was linked to greater personal growth (Ben-Yaakov & Taubman – Ben-Ari, 2021). Furthermore, personal growth three and six months after childbirth in first time mothers was also positively and significantly associated with COVID-19-related anxiety experienced during pregnancy (Navon-Eyal & Taubman – Ben-Ari, 2025).

Another study among first-time and non-first-time mothers following childbirth found that although there were no direct associations between adverse and benevolent childhood experiences and personal growth, there were significant indirect associations. Specifically, women who reported greater adverse and fewer benevolent childhood experiences tended to report higher distress, which in turn was associated with lower social support and lower self-compassion, and these in turn were associated with lower personal growth following childbirth (Chasson & Taubman – Ben-Ari, 2024).

According to the PTG model, prior traumatic experiences may challenge an individual’s core beliefs, such as their perceptions of the world’s fairness, the degree of control over their own life, and the meaning of their life (Tedeschi et al., 2018). These pre-existing experiences can disrupt established assumptions, heighten emotional distress (Calhoun & Tedeschi, 2013), and influence the potential for personal growth during the transition to motherhood.

### 1.5. Current Study

As discussed above, several studies have been conducted over the last decade using the PTGI in relatively small and limited samples of women following childbirth, examining various associations between background variables, stress-related aspects, and previous life events and personal growth. These studies have yielded equivocal, sometimes conflicting results. By combining the data from these studies into one large sample, we can re-examine these associations in a more robust way. In addition, the combined data offers an opportunity to investigate differences and similarities of these associations using different versions of the measurement tools, i.e., the original 21-item PTGI, its five domains, and the more recent 10-item PTGI-SF. Thus, the current cross-sectional study examines the associations of the following variables with PTG using these tools: demographics, i.e., age, education, economic status, religiousness, physical health, relationship status; perinatal-related aspects, i.e., infant age, infant birth weight, gestation week, kind of delivery, preterm or term, twins or single infant, fertility treatment, at-risk pregnancy, miscarriage; and experiencing stressful event in the previous year.

Research questions: (1) what are the associations between sociodemographic and perinatal-related variables, on the one hand, and PTGI, its five domains and the PTGI-SF, on the other?; and (2) are these associations similar when using the various PTGI scores (mean total scores of the long and short forms of the PTGI and the separate five growth domains scores)?

## 2. Methods

### 2.1. Participants

Table 1 presents the characteristics of the participants. The final sample consisted of 4641 women, mostly Jewish (95.8%), aged 18–49 (M = 29.43, SD = 4.82), who were first-time mothers of infants up to 24 months old (M = 6.82, SD = 4.42). Most of the participants were in a spousal relationship (95.9%); 73.5% had an academic degree, and the rest had a high school or post-high school diploma; 61.6% defined their income as average; 92.3% defined their health status as very good or good; and 47.5% identified as secular, with the rest identifying as traditional or religious.

### 2.2. Procedure

Each separate study received approval from the Institutional Review Board preceding data collection (n. 417006, June 2010; n. 142012, January 2012; n. 21808, March 2018; n. 012003, January 2020; n. 012107, February 2021; n. 082102, August 2021; n. 012205, January 2022; n. 012201, February 2022; n. 012303, January 2023). Most questionnaires were collected through an electronic link sent to relevant women via social media forums or through collaboration with physicians who recruited participants to the different studies. The inclusion criteria for the current study were being a first-time mother to a single infant or twin infants up to 24 months-old and being able to complete questionnaires in Hebrew. No other constraints were used for the sample. All women gave their informed consent to participate in the studies at the time they were conducted.

### 2.3. Instruments

**The Posttraumatic Growth Inventory** (PTGI; Tedeschi & Calhoun, 1996), adapted for mothers following childbirth (Taubman – Ben-Ari et al., 2011), was used to assess personal growth. It consists of 21 items across five domains: relations with others (7 items; Cronbach’s alpha 0.85); new possibilities in life (5 items; Cronbach’s alpha 0.76); personal strength (4 items; Cronbach’s alpha 0.83); spirituality (2 items; Cronbach’s alpha 0.74); and appreciation of life (3 items; Cronbach’s alpha 0.70). Participants were asked to rate the extent to which each change described had occurred for them since giving birth, using a 6-point scale from 0 (*I have not experienced this change*) to 5 (*I have experienced this change to a very great degree*). Growth scores for each domain were calculated as the mean of responses to the relevant items, with higher scores indicating greater perceived growth. Additionally, a total score was computed by averaging responses to all the items in the scale, with a Cronbach’s alpha of 0.90.

**The Posttraumatic Growth Inventory–short form** (PTGI-SF; Cann et al., 2010), was also used to assess personal growth. Participants were asked to indicate the degree to which they had experienced each of ten changes since childbirth (2 items for each of the five domains). The scale ranged from 0 (*I did not experience this change*) to 5 (*I experienced this change to a very great degree*). In the current study, Cronbach’s alpha was 0.84. Each participant’s score was calculated by averaging responses to the 10 items. Higher scores indicated a greater experience of personal growth.

Background characteristics were assessed using **a sociodemographic questionnaire**: age (continuous); education (1 = elementary; 2 = high school; 3 = post high school; 4 = academic); economic status (1 = below average; 2 = average; 3 = above average); physical health (1 = very poor; 2 = poor; 3 = average; 4 = good; 5 = very good); relationship status (1 = single; 2 = in a couple relationship); religion (1 = Jewish; 2 = Muslim; 3 = Christian; 4 = Druze; 5 = Atheist; 6 = Other); religiousness (1 = secular; 2 = traditional; 3 = religious; 4 = orthodox); a stressful event in the previous year (1 = yes; 2 = no); had a miscarriage (1 = no; 2 = yes); gestation week (continuous); at-risk pregnancy (0 = having medical diagnosis for a particular risk factor for pregnancy; 1 = regular/not at risk); fertility treatment (1 = spontaneous pregnancy; 2 = pregnancy following fertility treatment); kind of delivery (1 = vaginal; 2 = instrumental; 3 = c-section); preterm infant (1 = no; 2 = yes) (in studies that lacked the direct question regarding delivering preterm infant, the definition was delivery before week 36, or birth weight lower than 2000 g (Taubman – Ben-Ari et al., 2019); infant age (continuous); infant birth weight (continuous); number of babies (singleton or twins).

### 2.4. Data Analysis

Analyses were conducted using SPSS (ver. 24). Of the women, 3636 completed the original PTGI, and 1005 completed the PTGI-SF. As the ten items in the PTGI-SF are actually part of the original 21-item PTGI, we extracted these 10 items for all possible participants, so that a PTGI-SF score was calculated for all 4641 participants. It is important to note that not all participants had all background variables recorded, and thus the calculations are based on different numbers of participants. This information is indicated in the Tables.

First, we calculated the correlations between the PTGI scores and continuous background variables (participant’s age, education years, economic status, physical health status, religiousness, gestation week, infant age and weight). We then computed a series of ANOVAs to examine differences in the PTGI scores according to the study’s nominal variables: demographic information (being in a couple relationship or not), stress-related perinatal events (fertility treatment, previous miscarriage, at-risk pregnancy, type of delivery, pre-term childbirth), and stressful events in the previous year, controlling for the participants’ ages and the infants’ ages in all cases.

## 3. Results

### 3.1. Associations Between Background Variables and Personal Growth

The results of the Pearson correlations between the background variables and the original PTGI (21 items), its five domains, and the PTGI-SF (10 items) are presented in Table 2. As Table 2 shows, most of the correlations were rather weak, with younger age, fewer years of education, lower economic level, higher levels of religious observance and better health associated with higher levels of personal growth in the PTGI and the PTGI-SF. No significant correlations were found between infant age, weight and gestation week with the total scores of the PTGI and PTGI-SF. However, various associations were found relating to each of the five domains of PTGI and background variables. While age was associated with all domains, education and economic status were associated with four of the domains, but not with relationships with others; religiousness was associated with three of the domains, not including personal strengths and appreciation of life; and physical health was also associated with three domains, but not with spirituality and appreciation of life.

In addition, infant age, weight and gestation week had various associations with the five PTGI domains. Positive correlations were found between infant age and new opportunities, personal strengths and appreciation of life. Positive correlations were also found between infant weight and gestation week and spirituality, while negative correlations were found between gestation week and personal strengths and appreciation of life. 

### 3.2. Differences in PTGI, PTGI Domains and PTGI-SF According to Nominal Study Variables

Table 3 presents the means and standard deviations of the original PTGI, its five domains and the PTGI-SF, according to the study variables, along with the results of the ANOVAs. As can be seen from the table, higher personal growth (using both the PTGI and the PTGI-SF scores) was found among those not currently in a spousal relationship, those who had an at-risk pregnancy and those who had experienced a stressful event in the previous year. Only the PTGI-SF was higher among those who gave birth to twins and those who underwent fertility treatment. Only the PTGI was higher among those who had instrumental or c-section delivery than those who had a vaginal delivery. In addition, neither of the total scores were related to miscarriage or to preterm delivery.

Furthermore, in examination of the PTGI domains, perception of new opportunities, spirituality, personal strengths, and appreciation of life were higher among those not currently in a spousal relationship, those who had at risk pregnancies, and those who had experienced a stressful event in the previous year. The personal strength domain was higher among mothers of twins; the new opportunities in life domain was higher among women who underwent fertility treatments; new opportunities and personal strengths were higher for women with a history of miscarriage; personal strengths and appreciation of life were higher among mothers of preterm infants; and new opportunities and spirituality were higher among those who had instrumental or c-section deliveries.

## 4. Discussion

The current study utilizes a comprehensive dataset to examine the associations between various background variables and measures of personal growth following the transition to motherhood. By reviewing the past 15 years of research on this topic, alongside the present findings, we are able to address several key questions thoroughly. The data also provides more concrete insights regarding the usefulness of the long versus the short form of the PTGI within this population. Through this analysis, we can determine whether the long and short versions of the PTGI yield consistent associations with the background variables explored in this study, and whether the five growth domain scores offer additional value beyond the total scale scores.

The findings indicated that, in most cases, the PTGI and PTGI-SF had similar associations with the background variables, showing that age, education level and economic status were negatively related to personal growth, whereas religiousness and physical health were positively related to personal growth. In addition, both total scores were higher for mothers who were not in a spousal relationship, who carried at-risk pregnancy or who experienced a stressful event in the past year. Only the original PTGI score was higher for mothers who had an instrumental or c-section delivery; only the PTGI-SF score was higher for mothers of twins and those who underwent fertility treatment.

Regarding the PTGI domain scores, it appeared that in most cases the domains of personal strengths, spirituality, new opportunities, and appreciation of life had similar associations with the background variables as the two total scores, yielding significant connections with age, education level, economic status, not being in a spousal relationship, carrying an at-risk pregnancy and experiencing a stressful event in the past year. Other associations were more sporadic, but notably the domain of relating to others was associated with higher levels of religiousness and physical health, and delivery through instrumental or c-section procedures.

Of all the domains, new opportunities and personal strengths seemed to yield the highest number of associations with the examined background variables. Thus, higher perception of new opportunities following the transition to motherhood was related to lower age, education, and economic status, and to higher religiousness and physical health. In addition, higher perception of new opportunities was related to higher infant age, not being in a spousal relationship, carrying an at-risk pregnancy, a past experience of miscarriage, undergoing fertility treatments, an instrumental or a c-section delivery, and experiencing a stressful event in the past year.

Higher perception of personal strength following the transition to motherhood was related to lower age, education, and economic status, and to higher physical health. In addition, higher perception of personal strength was related to higher infant age, earlier gestation week, not being in a spousal relationship, carrying an at-risk pregnancy, having twins, giving birth prematurely, a past experience of miscarriage, and experiencing a stressful event in the past year.

Taken together, the findings validate previous research linking certain demographic and perinatal variables to personal growth (e.g., Ben-Yaakov & Taubman – Ben-Ari, 2021; Berman et al., 2021; Sawyer et al., 2015; Taubman – Ben-Ari et al., 2012; Noy et al., 2015; Rozen et al., 2018), while also showing new correlations, such as higher levels of personal growth among mothers of twins and those who underwent fertility treatments (e.g., Noy et al., 2015; Taubman – Ben-Ari et al., 2010). Moreover, the findings indicate that when comparing information gathered from the original PTGI and from the PTGI-SF, the latter proves to be highly effective, as it yields very similar results. We cannot conclude decisively that the PTGI domains provide more information than both total scores, as in most cases new opportunities and personal strengths were related to the study variables, and spirituality and appreciation of life were also related to a lesser degree. Overall, the results of the individual domains largely mirrored those of the total scores.

However, the scores for the separate domains may provide another kind of information, not available through both total scores, concerning specific growth aspects that may differ depending on the circumstances and concerning individual differences, both of which might be further explored in the future. This is highlighted by three examples from the current study for which differences were not found in the total PTGI/PTGI-SF score, as had been found in previous studies, but some domains of growth were found to be differentiated by those variables. Thus, having twins, undergoing fertility treatment, and the kind of delivery were found to be related to certain separate domains, providing a more nuanced understanding of the growth experience.

A key limitation of this study is that it only presents background information, and does not relate to psychological variables, either more cognitive variables such as the centrality of the event and ruminations (Tedeschi et al., 2018), or personality and emotional variables such as emotion regulation, optimism, or attachment orientations (Taubman – Ben-Ari, 2019). Future research should consider integrating these psychological variables, possibly by combining separate samples into a larger dataset, as in the present study. One should also acknowledge the fact that all the data was gathered in Israel, and thus might be potentially influenced by cultural norms, especially concerning religiosity, reproductive expectations, and the support systems available to new mothers. In addition, it is important to acknowledge the partial post hoc derivation of PTGI-SF scores from the 21-item PTGI. While this approach is acceptable and justified by item overlap, it may be to some extent a limitation in terms of construct validity, as participants were not instructed to respond using the short form. Finally, because of the large sample size, even minor effects may reach statistical significance and thus warrant cautious interpretation. Nevertheless, the findings are important providing direction for researchers and can also serve to enhance recent efforts to assist women in the perinatal period to experience personal growth, using various kinds of interventions, such as techniques of mindfulness (Ghaedi-Heidari et al., 2024) among others.

## 5. Conclusions

This study is significant as it allowed for the examination of multiple demographic and perinatal-related variables in a large sample of women transitioning to motherhood. As a result, the findings are more robust than those of the separate previous studies using relatively small samples, offering a clearer understanding of how these variables relate to personal growth. Additionally, the data suggests that regarding the various available measures of personal growth, there is not a clear advantage of one scale over another. Given the practical considerations of time constraints and questionnaire efficiency, we recommend using the shorter version of the PTGI for research purposes.

## Figures and Tables

**Table 1:** Sociodemographic and Background Characteristics.

Variable	
	***M (SD*** *)*
**Age** (*n* = 4538)	29.43 (4.82)
**Infant’s age** (month) (*n* = 4621)	6.82(4.42)
**Gestation week** (*n* = 4499)	38.85 (2.13)
**Infant’s weight at birth** (*n* = 1874)	3.09 (0.58)
	***n* (%)**
**Religiousness** (*n* = 4639)	
Secular	2202 (47.5)
Traditional	1135 (24.5)
Religious	1184 (25.5)
Orthodox	88 (1.9)
Other	30 (0.6)
**Relationship status** (*n* = 4636)	
Single	192 (4.1)
In a spousal relationship	4444 (95.9)
**Education** (*n* = 4567)	
Elementary	35 (0.8)
High school	565 (12.4)
Post-high school	608 (13.3)
Academic	3359 (73.5)
**Economic status** (*n* = 4579)	
Below average	780 (17.0)
Average	2820 (61.6)
Above average	979 (21.4)
**Physical health** (*n* = 4576)	
Very poor	17 (0.4)
Poor	46 (1.0)
Average	292 (6.4)
Good	1623 (35.5)
Very good	2598 (56.8)
**A stressful event in the past year** (*n* = 2637)	
No	1917(72.7)
Yes	720(27.3)
**Fertility treatments** (*n* = 4607)	
No	3801 (82.5)
Yes	806 (17.5)
**At-risk pregnancy** (*n* = 4294)	
Regular	3206 (74.7)
At risk	1088 (25.3)
Miscarriage (*n* = 2587)	
No	1979 (76.5)
Yes	608 (23.5)
**Kind of delivery** (*n* = 4630)	
Vaginal	3018 (65.2)
Instrumental	592 (12.8)
C-section	1020 (22.0)
**Preterm infant** (*n* = 3728)	
No	3467 (74.7)
Yes	257 (6.9)
**Singleton\twins** (*n* = 4639)	
Single baby	4456 (96.1)
Twins	183 (3.9)

**Table 2:** Pearson Correlations between Study Variables and the PTG Measures.

	PTGI	PTGI-Relations with Others	PTGI-New Opportunities	PTGI-Spirituality	PTGI-Personal Strengths	PTGI-Appreciation of Life	PTGI-SF
Age	−0.15 ***	−0.07 ***	−0.09 ***	−0.30 ***	−0.10 ***	−0.07 ***	−0.18 ***
Education	−0.15 ***	−0.00	−0.16 ***	−0.21 ***	−0.16 ***	−0.13 ***	−0.16 ***
Economic status	−0.11 ***	−0.03	−0.09 ***	−0.23 ***	−0.07 ***	−0.07 ***	−0.14 ***
Physical health	0.05 **	0.04 *	0.03 *	0.01	0.07 ***	0.02	0.05 ***
Religiousness	0.15 ***	0.08 ***	0.08 ***	0.45 ***	0.01	0.02	0.19 ***
Infant’s age	0.03	−0.03	0.08 ***	0.01	0.03 *	0.06 ***	0.02
Infant’s weight	0.00	−0.06	0.02	0.14 ***	−0.02	−0.03	−0.00
Gestation week	−0.02	0.00	−0.01	0.03 *	−0.05 **	−0.05 **	−0.02

* *p* ≤ 0.05, ** *p* ≤ 0.01, *** *p* ≤ 0.001. Note: N ranged between 3602 and 4538 for age; between 3562 and 4567 for education; between 3575 and 4579 for economic status; between 3571 and 4576 for physical health; between 3634 and 4639 for Religiousness; between 3622 and 4621 for infant’s age; between 883 and 1874 for infant’s weight; between 3504 and 4499 for gestation week. Bold is used to highlight significant correlations at *p* ≤ 0.001.

**Table 3:** Means, Standard Deviations, and F Scores for the PTG Measures by Study Variables.

		PTGI	PTGI-Relations with Others	PTGI-New Opportunities	PTGI-Spirituality	PTGI-Personal Strengths	PTGI-Appreciation of Life	PTGI-SF
In a spousal relationship								
No	*M*	3.53	3.13	3.5	2.89	4.07	4.20	3.68
	*SD*	0.07	0.09	0.09	0.13	0.09	0.08	0.06
								
Yes	*M*	3.18	2.97	2.98	2.09	3.78	3.96	3.29
	*SD*	0.01	0.02	0.02	0.03	0.02	0.02	0.01
								
	F (*p*-value)	22.78 ***	3.19	31.31 ***	35.37 ***	11.59 ***	8.77 **	36.59 ***
Twins								
No	*M*	3.19	2.97	2.99	2.12	3.78	3.97	3.30
	*SD*	0.01	0.02	0.02	0.03	0.02	0.02	0.01
								
Yes	*M*	3.30	3.01	3.14	2.14	4.00	4.10	3.43
	*SD*	0.06	0.08	0.08	0.12	0.08	0.07	0.06
								
	F (*p*-value)	3.10	0.22	3.27	0.05	8.43 **	3.68	4.29 *
Fertility treatments								
No	*M*	3.19	2.97	2.98	2.10	3.78	3.97	3.29
	*SD*	0.01	0.02	0.02	0.03	0.02	0.02	0.01
								
Yes	*M*	3.24	2.98	3.08	2.22	3.84	4.00	3.37
	*SD*	0.03	0.04	0.04	0.06	0.04	0.04	0.03
								
	F (*p*-value)	2.19	0.01	4.25 *	3.33	2.40	0.61	6.19 *
At-risk pregnancy								
No	*M*	3.17	2.98	2.96	2.12	3.75	3.94	3.28
	*SD*	0.02	0.02	0.02	0.03	0.02	0.02	0.01
								
Yes	*M*	3.30	2.97	3.14	2.34	3.94	4.10	3.43
	*SD*	0.03	0.04	0.04	0.05	0.03	0.03	0.03
								
	F (*p*-value)	14.56 ***	0.04	19.33 ***	13.78 ***	26.73 ***	20.31 ***	28.94 ***
Miscarriage								
No	*M*	3.20	2.97	3.00	2.12	3.81	4.00	3.30
	*SD*	0.02	0.02	0.02	0.03	0.02	0.02	0.02
								
Yes	*M*	3.27	2.98	3.14	2.17	3.90	4.06	3.36
	*SD*	0.03	0.04	0.04	0.06	0.04	0.03	0.03
								
	F (*p*-value)	3.70	0.07	9.24 **	0.64	4.19 *	2.62	3.21
Kind of delivery								
Vaginal	*M*	3.17	2.94	2.97	2.07	3.77	3.95	3.29
	*SD*	0.02	0.02	0.02	0.03	0.02	0.02	0.02
								
Instrumental	*M*	3.24	3.04	3.04	2.20	3.78	3.98	3.30
	*SD*	0.04	0.05	0.5	0.07	0.04	0.04	0.03
								
C-section	*M*	3.25	3.02	3.05	2.23	3.83	4.03	3.35
	*SD*	0.03	0.04	0.04	0.05	0.03	0.03	0.03
								
	F (*p*-value)	3.70 *	3.03 *	2.13	4.21 *	0.96	1.98	1.62
Preterm infant								
No	*M*	3.21	2.95	3.04	2.13	3.80	4.01	3.32
	*SD*	0.02	0.2	0.02	0.03	0.02	0.02	0.01
								
Yes	*M*	3.28	2.99	3.06	2.10	3.99	4.16	3.40
	*SD*	0.05	0.07	0.07	0.10	0.06	0.06	0.05
								
	F (*p*-value)	1.68	0.35	0.03	0.08	8.24 **	6.37 *	2.68
Stressful event in the past year								
No	*M*	3.11	2.98	2.85	2.04	3.68	3.81	3.25
	*SD*	0.03	0.03	0.03	0.05	0.03	0.03	0.02
								
Yes	*M*	3.25	3.05	3.02	2.23	3.82	4.02	3.38
	*SD*	0.04	0.05	0.05	0.07	0.04	0.04	0.03
								
	F (*p*-value)	9.64 **	1.52	9.24 **	5.50 *	7.02 **	17.42 ***	10.22 ***

* *p* ≤ 0.05, ** *p* ≤ 0.01, *** *p* ≤ 0.001; Note: N ranged between 137 and 184 for In a spousal relationship = no and between 3448 and 4334 for In a spousal relationship = yes; between 3431 and 4341 for twins = no and between 157 and 180 for twins = yes;; between 2951 and 3712 for fertility treatments = no and between 617 and 782 for fertility treatments = yes; between 2504 and 3131 for at-risk pregnancy = no and between 775 and 1061 for at-risk pregnancy = yes; 1978 for miscarriage = no and 608 for miscarriage = yes; between 2332 and 2955 for Kind of delivery = vaginal, between 471 and 580 for kind of delivery = instrumental, and between 779 and 982 for Kind of delivery = c-section; between 2486 and 3363 for preterm infant = no and between 207 and 252 for preterm infant = yes; between 1101 and 1830 for stressful event in the past year = no and between 502 and 699 for stressful event in the past year = yes. Bold is used to highlight significant effects at *p* ≤ 0.001.

## Data Availability

The datasets generated during the current study are available from the corresponding author upon reasonable request.
